# Cell Surface-Bound TIMP3 Induces Apoptosis in Mesenchymal Cal78 Cells through Ligand-Independent Activation of Death Receptor Signaling and Blockade of Survival Pathways

**DOI:** 10.1371/journal.pone.0070709

**Published:** 2013-07-24

**Authors:** Christina Koers-Wunrau, Corinna Wehmeyer, Anja Hillmann, Thomas Pap, Berno Dankbar

**Affiliations:** Institute of Experimental Musculoskeletal Medicine, University Hospital Muenster, Muenster, Germany; University of Illinois at Chicago, United States of America

## Abstract

**Background:**

The matrix metalloproteinases (MMPs) and their endogenous regulators, the tissue inhibitor of metalloproteinases (TIMPs 1–4) are responsible for the physiological remodeling of the extracellular matrix (ECM). Among all TIMPs, TIMP3 appears to play a unique role since TIMP3 is a secreted protein and, unlike the other TIMP family members, is tightly bound to the ECM. Moreover TIMP3 has been shown to be able to induce apoptotic cell death. As little is known about the underlying mechanisms, we set out to investigate the pro-apoptotic effect of TIMP3 in human mesenchymal cells.

**Methodology/Principal Findings:**

Lentiviral overexpression of TIMP3 in mesenchymal cells led to a strong dose-dependent induction of ligand-independent apoptosis as reflected by a five-fold increase in caspase 3 and 7 activity compared to control (pLenti6/V5-GW/lacZ) or uninfected cells, whereas exogenous TIMP3 failed to induce apoptosis. Concordantly, increased cleavage of death substrate PARP and the caspases 3 and 7 was observed in TIMP3 overexpressing cultures. Notably, activation of caspase-8 but not caspase-9 was observed in TIMP3-overexpressing cells, indicating a death receptor-dependent mechanism. Moreover, overexpression of TIMP3 led to a further induction of apoptosis after stimulation with TNF-alpha, FasL and TRAIL. Most interestingly, TIMP3-overexpression was associated with a decrease in phosphorylation of cRaf, extracellular signal-regulated protein kinase (Erk1/2), ribosomal S6 kinase (RSK1) and Akt and serum deprivation of TIMP3-overexpressing cells resulted in a distinct enhancement of apoptosis, pointing to an impaired signaling of serum-derived survival factors. Finally, heparinase treatment of heparan sulfate proteoglycans led to the release of TIMP3 from the surface of overexpressing cells and to a significant decrease in apoptosis indicating that the binding of TIMP3 is necessary for apoptosis induction.

**Conclusion:**

The results demonstrate that exclusively cell surface-bound endogenous TIMP3 induces apoptosis in mesenchymal Cal78 cells through ligand-independent activation of death receptor signaling and blockade of survival signaling pathways.

## Introduction

TIMPs are the natural protease inhibitors of MMPs, which belong to a family of endopeptidases. The four TIMP members (1–4) are relatively small proteins of 21 to 28 kDa molecular mass. They are mainly responsible for the physiological remodeling of the ECM by maintaining the balance between matrix destruction and formation. An imbalance between MMPs and TIMPs leads to excess MMP activity and is associated with ECM degradation in various inflammatory conditions and in malignant tumors [1], [2], where the proteolytic turnover of basement membrane and ECM by MMPs is an important event in tumor growth, invasion and metastasis [3].

Among all TIMPs, TIMP3 plays a unique role. TIMP3 is a secreted protein and, unlike the other TIMP family members, tightly bound to the ECM, suggesting that TIMP3 activity is confined mainly to the cell surface [4]. TIMP3 is sequestered to the ECM in both its glycosylated 27 kDa and unglycosylated 24 kDa form, interacting with the ECM via both its N- and C-terminal domains [5]. Some observations suggest that TIMP3 is bound to negatively charged molecules such as heparan sulfate and other sulfated glycosaminoglycans although the specific function of TIMP3 bound to the ECM or to the cell surface is not yet known [6]. Beside its MMP inhibitory property [7], TIMP3 is able to serve as an inhibitor of several members of the adamalysin family, the adamalysin metalloproteinases with a disintegrin and metalloproteinase domain (ADAM) and ADAM with thrombospondin-like domains (ADAM-TS) [8]–[10], known to be involved in the shedding of cell surface molecules e.g. receptors, proteoglycans, adhesion molecules [11]–[13]. Thus, the huge amount of molecules affected by TIMP3 may reflect its broad range of cell regulatory functions such as proliferation, migration, invasion, differentiation, and apoptosis [1], [14]–[16]. Among all, the most interesting features of TIMP3 are the inhibition of tumor cell invasion and the potent proapoptotic effect on tumor cells *in vivo* [14], [17]–[21]. In accordance to this, expression of TIMP3 is silenced in various types of malignant cells [22]. Several studies have demonstrated the ability of TIMP3 to induce apoptosis in mammalian cells by stabilization of the cell surface death receptors tumor necrosis factor-receptor I (TNF-RI), FAS (CD95) and TNF-related apoptosis-inducing ligand-receptor I (TRAIL-RI), thereby increasing the susceptibility to ligand-induced apoptosis [23]–[25]. In this context, it has been shown that death receptor signaling is suppressed in many types of tumor cells by silencing death receptor and caspase-8 genes [26], [27].

In the present study, we show that lentiviral overexpression of TIMP3 in mesenchymal cells leads to both an increase in ligand-dependent as well as ligand-independent apoptosis. Moreover, we demonstrate that apoptosis in the absence of death ligands is associated with an inhibition of mitogen-activated protein kinase (MAPK)/ERK and Akt signaling pathways. However, most importantly, we highlight for the first time that binding of TIMP3 to cell surface proteogylcans is required for the ligand-independent proapoptotic effect of TIMP3.

## Materials and Methods

### Cell Lines

The human chondrosarcoma cell line Cal78 (Leibniz Institute DSMZ-German Collection of Microorganisms and Cell Cultures, Braunschweig, Germany) was cultured in RPMI. The cell line 293FT (Invitrogen, Karlsruhe, Germany) for producing lentiviral stocks were maintained in DMEM. The high resistance C7 subclone of Madin-Darby canine kidney (MDCK-C7) cells that were used in the MATRIN assay were cultured in MEM. If not indicated otherwise all media were supplemented with 10% FCS and 100 U/ml penicillin and 100 µg/ml streptomycin. Cell images were taken using an AxioVert.A1 microscope with an AxioCam digital camera and AxioVision software (Zeiss, Jena, Germany).

### Lentiviral Construct

For gene transfer, the human TIMP3 gene was directionally cloned in the lentiviral pLenti6/V5-D-TOPO (Invitrogen) containing a human cytomegalovirus immediate early promoter for high-level constitutive expression of TIMP3 (TIMP3-V5). The lentiviral vector pLenti6/V5-GW/lacZ serves as a positive control (LacZ-V5) (Invitrogen). Both vectors control C-terminal V5 epitope for detection of overexpressed TIMP3 or ß-galactosidase as an expression control.

### Lentiviral Infection

Lentiviral infection was performed as described in ViraPower™ Lentiviral Expression Systems (Invitrogen). In short, the expression construct and an optimized packaging mix were cotransfected into 293FT cells. After harvesting lentiviral supernatants, the cell line Cal78 were stable transduced. After selection different positive single cells were picked and expanded. Selections for stably transduced cells were achieved by BlasticidinS for 14 days (Invitrogen). Protein overexpression by selected clones was analysed after 72 hours (h) by western blot.

### MATRIN Assay

For the determination of the invasiveness of transduced Cal78 cells the cell-based matrix-associated transepithelial resistance invasion (MATRIN) assay was performed as described previously [28]. In short, an epithelial MDCK–C7 cell monolayer that develops a high transepithelial electrical resistance (TEER) was grown on the reverse side of filter cups (Heidelberg, Germany) within six-well dishes. TEER across the MDCK-C7 monolayer was measured with a set of two circular Ag/AgCl electrodes connected with an Ohm-meter (World Precision-Instruments, Sarasoto, USA). The filter membranes were additionally coated with a collagen layer using PureCol (Inamed Research GmbH, Gauting, Germany). The MDCK-C7 monolayers were grown until TEER had reached more than 4000 Ω before Cal78 were added into filter cups. Disturbing MDCK-C7 cell monolayer integrity by Cal78 leads to a decrease of the TEER. All measurements were performed in quadruplicate and terminated after total breakdown of the electrical resistance.

### Cell Adhesion

Cell adhesion was designated by CyQuant assay (Invitrogen, Karlsruhe), according to the manufactureŕs specifications. Cells were seeded in 96 well plates coated with purified collagen type-I (Cell Concepts, Umkirch) or fibronectin (Chemicon, Temecula, USA). We performed three independent experiments in triplicates.

### Antibody Array Kit

For the determination of the relative levels of phosphorylation of MAPK we used the Human Phospho-MAPK Array Kit (R&D Systems) in accordance with the manufacturers.

### Western Blotting

Cells were scraped and lysed in NP40 buffer supplemented with Protease Inhibitor Cocktail (Roche Diagnostics, Mannheim, Germany) for 1 h at 4°C. Protein concentration was quantified using the DC protein assay kit (Biorad). The supernatants of 4000 cells or cell lysates (30 to 80 µg protein) were boiled in Laemmli buffer containing ß-mercaptoethanol. Probes were resolved by 12% or 15% SDS-PAGE and, after TANK blot onto a PVDF membrane. These membranes were blocked by 3% BSA or 5% milk in TBS and washed in TBS addition of 0.05% TWEEN. The proteins were detected with appropriate antibodies using the ECL detection system (GE Healthcare, Munich, Germany). For heparinase experiments total cell lysates or supernatants digested for 4 h were used for western blot as described above. For the quantification of western blots we used Photoshop CS3.

### Antibodies

The antibodies used for western blotting were V5 (Invitrogen) and V5-HRP (Invitrogen), caspase-8 (Santa Cruz), caspase-9 (Cell Signaling), phospho-p44/42, p44/42, phospho-c-Raf, phospho-Akt, Akt, RSK1 and GAPDH-HRP, cleaved caspase-3 and -7, cleaved poly (ADP-ribose) polymerase (PARP) (Cell Signaling), beta actin (Sigma-Aldrich, Muenchen), LC3 (Nanotools), Beclin (Cell Signaling) and TIMP3 (Affinity BioReagents, Golden, CO) were used in the dilution according to the manufacturers’ specifications.

### Cell Death

Apoptotic cell death of 10.000 cells in a 96 dish was quantified using the Cell death detection ELISA (Roche) and additionally, the Apo-ONE Homogeneous caspase-3/7 Assay both according to the manufacturerś instructions. Therefore 8000 cells were seeded on 96-well plates and incubated for indicated periods of time. Apoptosis was evaluated using the caspase-3/7 assay after 41 h with or without administration of 100 ng/ml TNF-alpha (TNF-a), 100 ng/ml Fas-ligand (FasL) (R&D-Systems) or 100 ng/ml TRAIL (Pepro Tech, Rocky Hill, NJ) for 16 h. In some experiments Cal78 cells were treated with 25 to 200 nM recombinant human (rh) TIMP3 (R&D-Systems) for 96 h. For stimulation experiments cells were treated with 100 ng/ml rh epidermal growth factor (EGF), transforming growth factor-beta (TGF-b) or fibroblast growth factor-2 (FGF) (R&D-Systems) for 24 h and under withdrawal of serum (2% or 1% FCS) or ITS media (Sigma-Aldrich). Cal78 or Cal78 transduced with LacZ or TIMP3 were cultured and subsequently treated with heparinase I and III (Sigma-Aldrich). For the activation of heparinises 4 mM CaCl_2_ was added. Caspase-3 and -7 activities were measured after 72 h. The apoptosis response of the different cell clones was analysed by western blotting.

### Statistical Analysis

Statistical analysis was performed using GraphPad Prism Software, 5.0a (Graph Pad Software Inc., San Diego, CA, USA). Differences between groups were examined for statistical significance using Mann-Whitney test, and a value of less than p<0.05 was considered statistically significant.

## Results

### Transgene Production and Functionality of TIMP3

**Figure 1:**
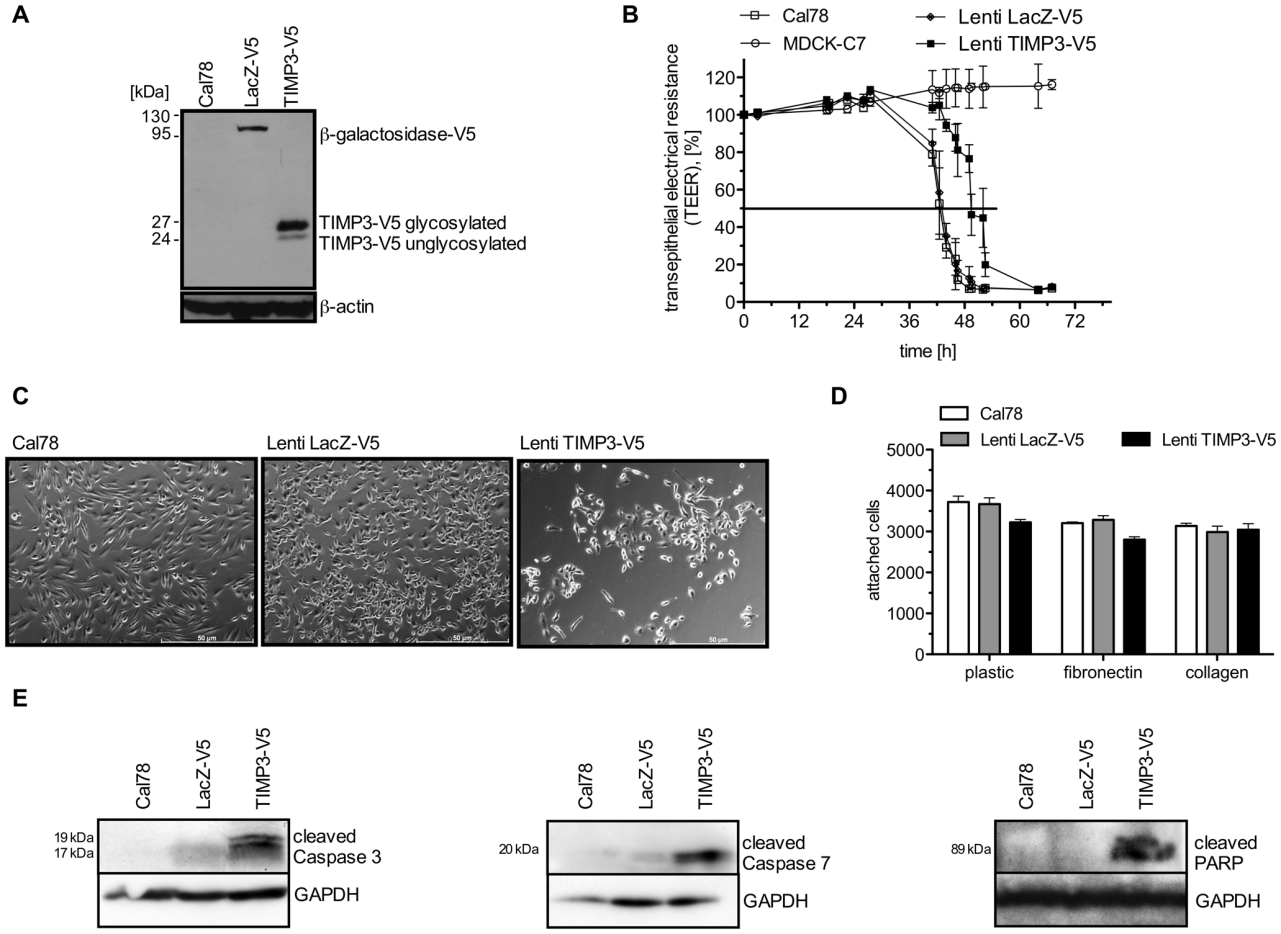
Transgene production and functionality of TIMP3. **A:** Expression of recombinant proteins was determined by Western Blot. Cal78 cells were either left untransduced (lane 1) or transduced with the control lentivirus (Lenti LacZ-V5, lane 2) or with Lenti TIMP3-V5 (lane 3) as indicated. Total cell lysates were analyzed for V5-tagged TIMP3 expression. **B:** Activity of lentiviral-expressed TIMP3 was confirmed by matrix-associated transepithelial resistance invasion (MATRIN) assay. The invasion capacity of untransduced CAL78 (square) or cells transduced Lenti LacZ-V5 (black triangle) or Lenti TIMP3-V5 (black square) was determined as transepithelial electrical resistance (TEER). MDCK-C7 cells served as an internal control (circle). **C:** Images of Cal78 cultures (left top), CAL78 transduced with the control plasmid LacZ-V5 (right) or with Lenti TIMP3-V5 (left bottom). The scale bar represents 50 µm. **D:** Effect of TIMP3 overexpression on cell adhesion on plastic, fibronectin and collagen surfaces 4 h after plating the cells. **E:** Cleavage of caspase 3, 7 and PARP in Cal78 cells and transduced Cal78 cells with LacZ or TIMP3 was determined after 72 h by western blot analyses.

To investigate the apoptotic effect of TIMP3, the mesenchymal cell line Cal78 was stably transfected with a lentiviral expression construct containing full-length human TIMP3. Evidence that TIMP3 and the control protein ß-galactosidase were overexpressed by lentiviral transduced Cal78 cells is given in figure 1A. Both the unglycosylated and glycosylated form of TIMP3 were detectable in extracted proteins from whole cell lysates with higher levels of the glycosylated form found in comparison to the unglycosylated form (figure 1A).

Since TIMP3 has been shown to inhibit the invasion in various cell types [1], [29], MATRIN assay measurements were performed to test whether the lentiviral expressed TIMP3 is functionally active. As shown in Figure 1B, addition of untransduced and LacZ-transduced Cal78 cells resulted in a 50% breakdown of the transepithelial electrical resistance (TEER) after 43 h. Cal78 reached a complete breakdown (10% of TEER) after 48 h and LacZ-transduced Cal78 cells after 50 hours. However, addition of TIMP3 transduced cells led to a delayed decline in TEER, as reflected by a 50% breakdown after 50 hours, followed by a complete breakdown after 60 h, indicating a significantly lower invasive capacity of TIMP3-overexpressing cells than corresponding control cells and hence the expression of functionally active TIMP3 (figure 1B). MDCK-C7 cells were able to keep the initially established TEER for more than 67 hours, demonstrating that the lack of cells in the upper compartment preserved the integrity of the monolayer over prolonged periods of time.

Analyses of permanent cultures of transduced cells revealed that overexpression of TIMP3 causes morphological alterations. After 72 h, most cells expressing TIMP3 became profoundly rounded, bigger in size and refractile in cell shape accompanied by partial detachment of cells when compared with untransduced or LacZ transduced cells (figure 1C). In order to determine if the loss of adherence is a consequence of cell death or depends on general adhesion defects caused by TIMP3 overexpression, we analyzed the adhesion capability early after cells were transferred to plastic or coated surfaces. There were no obvious differences in the number of adherent cells between lentiviral TIMP3 transduced cells and the corresponding controls cultured on the different surfaces (figure 1D), indicating that the loss of adherence is a consequence of apoptotic cell death. To examine in more detail, whether the observed effects in Cal78 cells are a consequence of apoptosis induced by lentivirally delivered TIMP3, we studied the cleavage of PARP and caspase-3 and -7. Increased cleavage of death substrate PARP was noted in TIMP3 overexpressing cultures, indicating activation of caspase-3. Indeed, Western Blot analyses confirmed the activation of caspase-3 and -7 by detecting the cleaved forms of both caspases in TIMP3 overexpressing Cal78 (figure 1E).

### TIMP3 Overexpression Induces Apoptotic Cell Death

**Figure 2:**
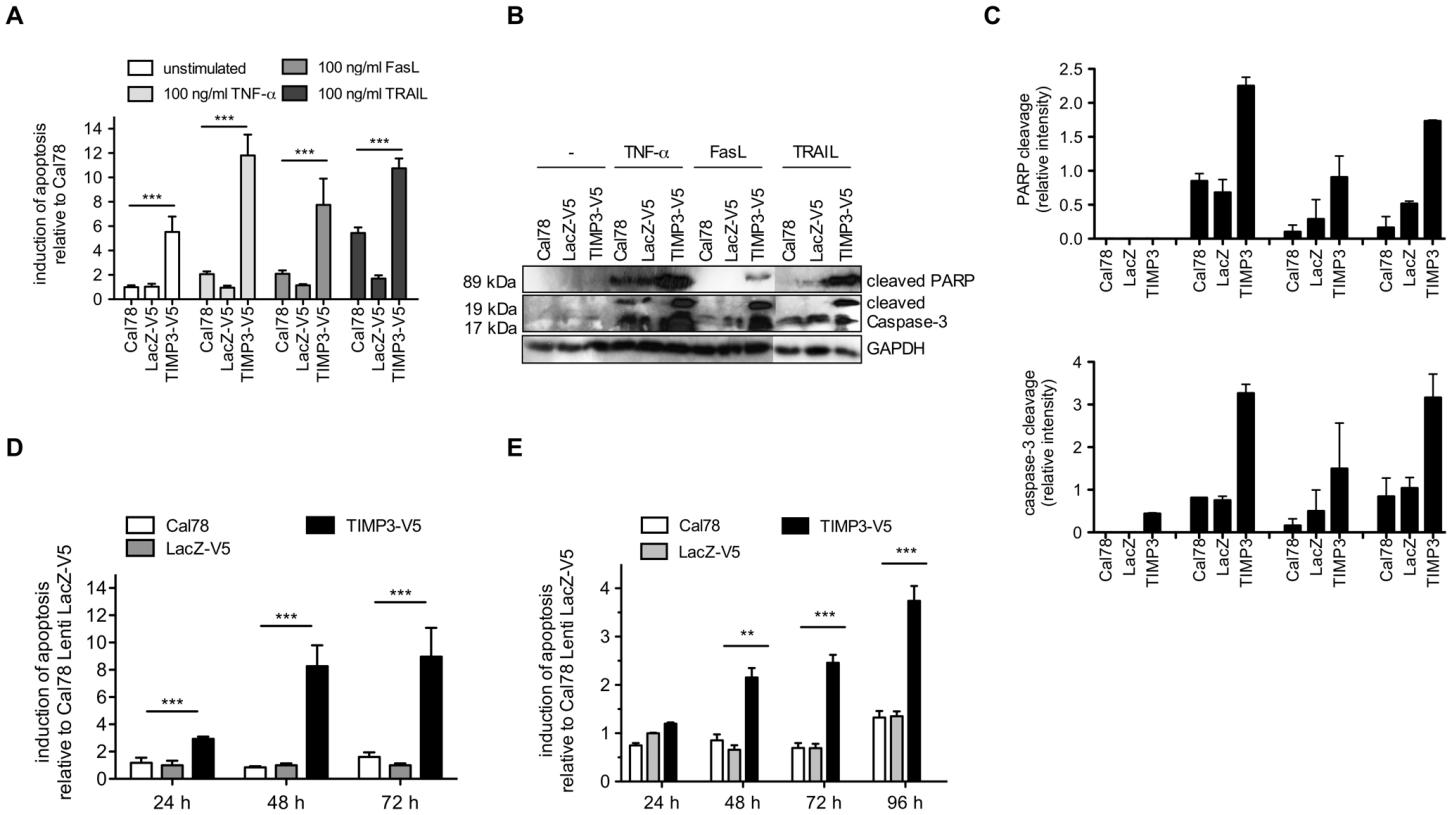
Effect of TIMP3 on apoptosis. **A:** Death receptor dependent apoptosis was analyzed in Cal78 cells and Cal78 cells transduced with LacZ or TIMP3 cultured for 24 h and stimulated with 100 ng/ml FasL, TNF-a or TRAIL for 16 hours by measurement of caspase 3 and 7 activities and **B,C:** cleavage of caspase 3 and PARP in western blot analyses. GAPDH serves as an internal control. **D:** TIMP3-induced apoptosis was evaluated in Cal78 cells and transduced Cal78 cells with LacZ or TIMP3 cultured for 24 to 72 h. Apoptosis was assessed by measurement of caspase 3 and 7 activities and **E:** by histone fragmentation assay.

In order to investigate the proapoptotic effect of lentiviral overexpressed TIMP3, we first studied whether TIMP3 induces death receptor dependent apoptosis, reflected by an increased susceptibility to apoptosis induced by death receptor ligands. For this purpose, Cal78 cells were first cultured for 24 h, followed by incubation with 100 ng/ml each of TNF-α, FasL, or TRAIL for 16 h. Stimulation of TIMP3 overexpressing cells with TNF-a, FasL and TRAIL-induced a high and significant increase in apoptosis (figure 2A) relative to untransduced and control transduced cells (11.8-fold for TNFa, 7.7-fold for FasL, and 10.8-fold for TRAIL). In contrast, all death receptor ligands only marginally induced apoptosis in untransduced cells, while hardly any induction in the LacZ-transduced cells was observed. In line with these findings, stimulation of TIMP3 overexpressing cells with TNF-a, FasL and TRAIL leads to the cleavage of caspase-3 and increased levels of cleaved death substrate PARP (figure 2B,C). Likewise, all death receptor ligands only slightly induced cleavage of caspase-3 and PARP in untransduced cells.

**Figure 3:**
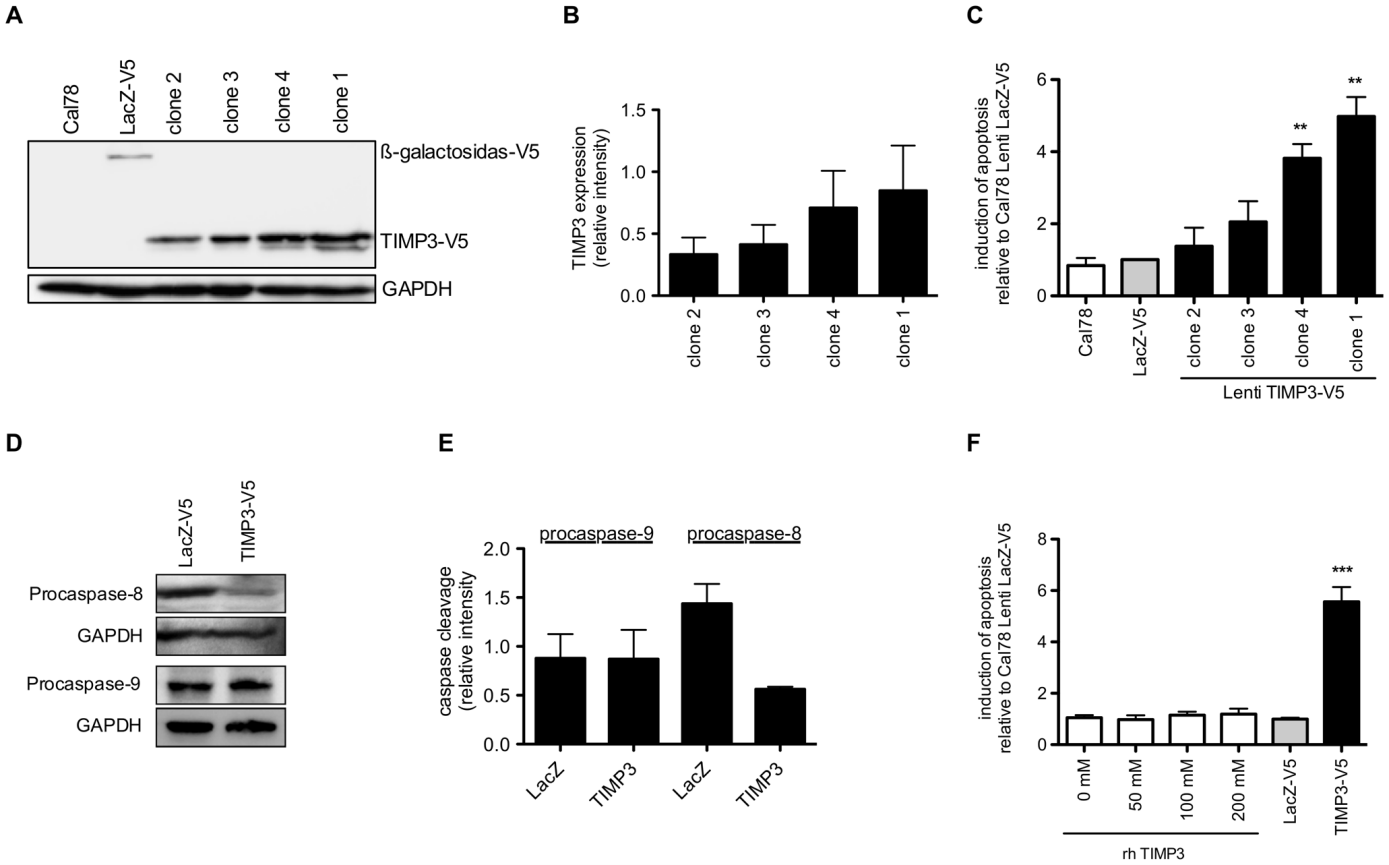
Dose-dependent effect of TIMP3 on apoptosis. **A–C:** Determination of the apoptosis response in different cell clones (clone 1 to 4) by caspase-3/7 activity and corresponding TIMP3 expression in these cell clones. **D,E:** Activation of initiator caspases-8 and -9 in transduced Cal78 cells with LacZ or TIMP3 cultured for 72 h. **F:** The effect of exogeneous TIMP3 on apoptosis after stimulation of Cal78 cells with recombinant human (rh) TIMP3 up to 200 nM for 96 h. Values less than p<0.05 (*) were considered statistically significant.

Intriguingly, in the absence of death receptor ligands, overexpression of TIMP3 alone led to a strong induction of apoptosis reflected by a 5.5-fold increase relative to Cal78 cells transduced with LacZ-V5. To investigate this spontaneous induction of apoptosis by TIMP3 in detail, we analyzed apoptosis rates after 24, 48 and 72 h of culture relative to cells transduced with the control vector. Caspase-3 and -7 activity increases from 2.9-fold after 24 h to 9-fold after 72 h (figure 2D) and histone fragmentation increases over a time course of 96 h up to 3.5-fold in TIMP3 overexpressing cells (figure 2E) indicating a considerable increase in cells undergoing apoptosis over the time. Moreover, analyses of different TIMP3 overexpressing clones demonstrated a clear correlation of lentiviral TIMP3 protein and ligand-independent apoptosis (figure 3A–C).

Based on these data, we investigated whether overexpression of TIMP3 results in the activation of caspase-8, as a key mediator in death receptor-mediated signaling, or in the activation of caspase-9, which is linked to the mitochondrial pathway. Activation of caspase-8 but not caspase-9, detected as a reduction in the levels of their proforms, was observed in TIMP3-overexpressing cells, indicating activation of death receptors without addition of death receptor ligands (figure 3D,E).

Next, we asked whether recombinant TIMP3 (rhTIMP3) has a similar effect on the induction of apoptosis. In this context Ahonen *et al.* show that 50 nM rhTIMP3 has an effect on different melanoma cell lines [23]. Notably, stimulation of mesenchymal Cal78 cells with up to 200 nM TIMP3 for 96 h revealed no induction of caspase-3 and -7 activity (figure 3F), implicating that exogenous rhTIMP3 is not able to induce apoptosis in these cells.

### Cell Surface Binding of TIMP3 is Required for Apoptosis Induction

**Figure 4:**
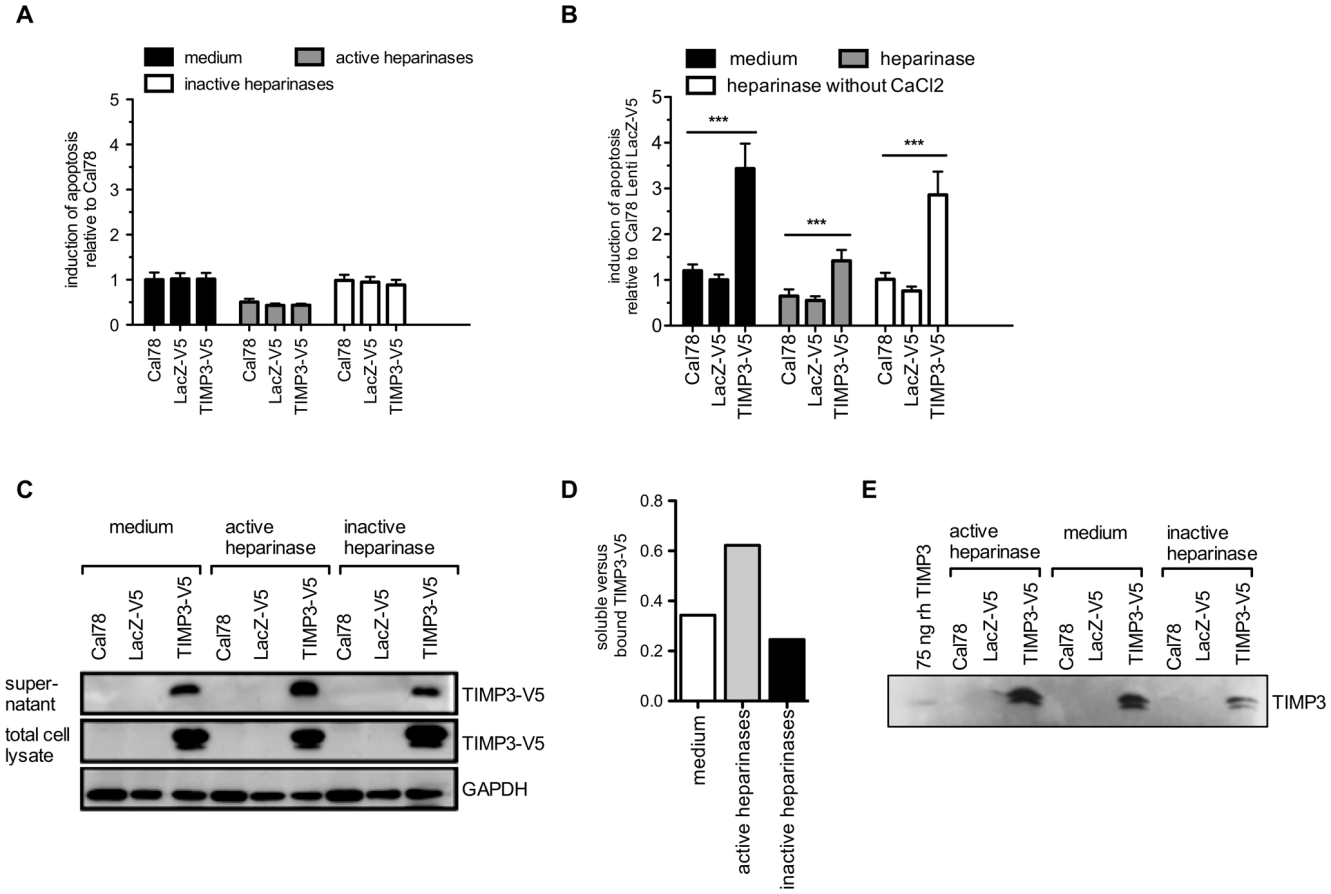
Proteoglycan-bound TIMP3 induces apoptosis. **A:** Cal78 or Cal78 transduced with TIMP3 or LacZ were cultured and subsequently treated with heparinase I and III for 2 hours. Supernatants from non-treated and treated cells were transferred onto Cal78 cells for 72 hours prior assessment of apoptosis **B:** Cal78 or Cal78 transduced with TIMP3 or LacZ were incubated with or without heparinase I and III for 72 hours until evaluation of apoptotic cell death. Inactive heparinases (without CaCl_2_) were used as a treatment control. Apoptosis was assessed by measurement of caspase 3 and 7 activities. Values less than p<0.05 (*) were considered statistically significant. **C:** Evaluation of TIMP3 release from cell surface of transduced cells by heparinase. Western Blot analysis of TIMP3-V5 from cell extracts and corresponding supernatants 4 hours after treatment with heparinase. **D:** Quantification of the Western Blot bands of figure 3C. The band of soluble TIMP3-V5 from supernatants was shown versus bounded TIMP3-V5 from the cell lysates. **E:** Western blot analysis of the supernatants shown in figure C with a specific TIMP3 antibody. 75 ng/ml rh TIMP3 serves as an indication of the amount of TIMP3 in supernatants of Cal78 cells.

Although TIMP3 has been described to be a mainly matrix-associated protein, TIMP3 was also detectable in the supernatant of overexpressing cells (figure 4C and 4E). In order to explore a possible bystander effect of soluble native TIMP3, supernatants of TIMP3 transduced cells were transferred onto uninfected Cal78 cells for 72 h. None of the supernatants were able to trigger apoptosis in untransduced cells (figure 4A, black bars). Since it has been demonstrated that TIMP3 binds to heparan sulfates and can be released from the matrix by heparinase [6], TIMP3 transduced cells were treated with heparinase for further enrichment of TIMP3 in the supernatants. Indeed, a higher amount of TIMP3 was detected in supernatants of cells treated with active heparinase compared to treatment with inactive enzyme or untreated cells (figure 4C–D). However, supernatants of heparinase-treated cells, despite high levels of TIMP3 (figure 4C and 4E), were still not able to induce apoptosis in Cal78 cells (figure 4A, grey and white (control) bars), suggesting that binding of TIMP3 to the cell surface is required for TIMP3-mediated induction of apoptosis. To substantiate this concept, again TIMP3 was eliminated from the cell surface of overexpressing cells by heparinase digestion of proteoglycans and induction of cell death was determined. Overexpression of TIMP3 caused a 2.9-fold induction of apoptosis compared to untransduced Cal78 cells. However most importantly, treatment with heparinase resulted in a significant decrease in apoptosis (1.2-fold induction of apoptosis), whereas treatment of transduced cells with non-active heparinase (without addition of CaCl_2_) revealed similar apoptosis values than non-treated controls (2.4-fold induction of apoptosis) (Figure 4B).

### TIMP3-induced Apoptosis is Influenced by Serum Factors

**Figure 5:**
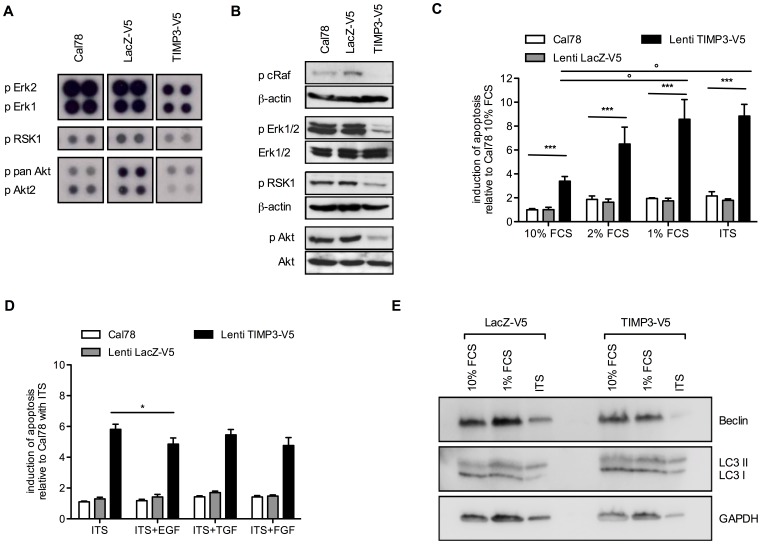
TIMP3-induced apoptosis is influenced by serum factors. **A:** Influence of TIMP3 on activation of cRaf, ERK1/2, RSK1 and Akt was determined in Cal78 cells and transduced Cal78 cells with LacZ or TIMP3 by spot array measurements. **B:** Phosphorylation of cRaf, ERK1/2, RSK1 and Akt was confirmed by western blotting. **C:** Influence of serum withdrawal on apoptosis rates after 24 hours. Apoptosis in lentiviral transduced cells was measured relative to Cal78 cells cultured with 10% FCS. Values less than p<0.05 (*or °) were considered statistically significant. **D:** Influence of specific growth factors on apoptosis rates under serum-free conditions. Prior to the assessment of apoptosis, Cal78 cells and transduced Cal78 cells with LacZ or TIMP3 were stimulated with 100 ng/ml EGF, TGF-ß or FGF-2 and cultured for 24 hours. Apoptosis was defined by caspase 3 and 7 activities. *Indicates statistical significance (p<0.05). **E:** Influence of serum withdrawal on autophagocytosis in Cal78 cells after 24 hours. Autophagocytosis in TIMP3 overexpressing CAL78 cells was determined relative to Cal78 cells transduced with a control construct (LacZ) cultured with 10% FCS, 1% FCS or ITS by Western blot analysis of the typical marker proteins LC3 and Beclin.

Since it has previously been demonstrated that ERK and Akt signaling have protective effects against death receptor-induced apoptosis [30], we analyzed the MAPK pathway in TIMP3-induced apoptosis in Cal78 cells by different MAPK spot arrays. The comparison of lentiviral transduced TIMP3 cells with the control cells and cells transduced with the LacZ vector showed a decrease in phosphorylated ERK1 and ERK2 as well as RSK1, a downstream target of ERK (figure 5A). To confirm these data, phosphorylation of ERK1/2, RSK1, and additionally cRaf, a protein upstream of ERK as well as Akt, a member of the PI3K pathway were analyzed by western blotting. In accordance to the results from the phosphorylation arrays, all tested signaling pathway components were less activated after lentiviral TIMP3 transduction (figure 5B).

In this context, we found that withdrawal of serum for 24 h led to a further enhancement of TIMP3-induced apoptosis. The reduction of serum from 10% to 1% FCS caused a significant increase in the apoptosis trigger from 3.4-fold up to 8.6-fold in TIMP3 transduced Cal78. Moreover, no further increase was observed when serum-free medium, supplemented with insulin, transferrin, and selenium (ITS) (8.8-fold) was used instead of 1% FCS (figure 5C). These results suggest that serum-derived survival factors influence TIMP3-induced apoptosis. In order to verify an influence of survival factors, we tested the effects of EGF, TGF-ß and FGF-2 on TIMP3-induced apoptosis. Administration of EGF resulted in a significant reduction of the apoptosis response in TIMP3 transduced Cal78 cells, whereas FGF-2 and TGF-ß have minor or no effects, respectively (figure 5D). In order to proof whether serum deprivation initiates autophagy in these cells, which facilitates the apoptosis induction, we analysed levels of the autophagy marker proteins LC3 and Beclin. There was no significantly enlarged conversion of LC3I to LC3II in cells overexpressing TIMP3 in contrast to control cells. Furthermore, there were equal amounts of Beclin in comparable cultured cells (figure 5E), excluding autophagy in these cells.

## Discussion

Evidence from several studies shows that overexpression of TIMP3 inhibits tumor growth *in vivo* [31]–[33]. The role of TIMP3 as a putative tumor suppressor is confirmed by the fact that TIMP3 is silenced in various types of human cancers and malignant cell lines [22], [34], [35], suggesting that the silencing of TIMP3 in tumor cells is an important event during tumor development. Moreover, recent studies in mice lacking TIMP3, further extend the role of TIMP3 as a tumor suppressor and emphasize TIMP3 as a candidate for therapeutic use in cancer [36], [37].

In the present study we investigated the proapoptotic effect of TIMP3 on the mesenchymal cancer cell line Cal78. For this purpose, we used a lentiviral expression system to generate stably transduced TIMP3-overexpressing tumor cell clones. As expected, lentiviral transduced cells produced persistently high levels of TIMP3. In order to approve activity of lentivirally expressed TIMP3, we used our previously established invasion assay (MATRIN, [28]) to assess whether TIMP3 is able to inhibit MMP-mediated cell invasion. In accordance with studies demonstrating an inhibitory effect of TIMP3 on the invasion of different invasive cancer cells [19], lentiviral overexpression of TIMP3 significantly inhibited the invasiveness of Cal78 cells, indicating the expression of functional active TIMP3.

Several studies have shown that TIMP3 is able to induce apoptosis in different cell types including retinal pigment epithelial cells, melanoma and prostate cancer cell lines [14], [18], [19], [38], [39]. Moreover, a recent animal study has demonstrated that deficiency of TIMP3 leads to apoptotic cell death in involuting breast tissue [40]. Consistently, assessment of apoptosis in TIMP3 overexpressing Cal78 revealed a significant induction of apoptosis by the death receptor ligands FasL, TNF-a, and TRAIL, suggesting that lentiviral-mediated overexpression of TIMP3 also leads to an inhibition of the shedding of death receptors. In addition to this, it has been described that TIMP3 by itself is able to induce apoptosis. Although some studies indicated that exogenous recombinant human TIMP3 causes apoptosis [14], [19], [23], we did not observe any proapoptotic effect of recombinant TIMP3 on mesenchymal Cal78, even at higher concentrations than previously described to be effective [19]. Likewise, supernatants obtained from TIMP3 overexpressing Cal78 cells were not able to induce apoptotic cell death in non-transduced cells, further confirming that the soluble form of TIMP3 does not have the capability to inducing apoptosis. Moreover, the same authors demonstrated that adenovirally overexpressed TIMP3 inhibits adhesion of melanoma cells to ECM prior to induction of apoptosis [18]. In our hands, overexpression of TIMP3 had no influence on the adhesion behavior of Cal78 cells neither on uncoated nor on collagen type 1 or fibronectin coated surfaces after 4 h, suggesting that the loss of adhesion observed at later time points is rather a consequence of apoptosis than a preceding adhesion defect caused by TIMP3. The apparent discrepancies in the TIMP3 mediated effects may be explained by the considerable differences between the highly aggressive melanoma cell line used by Ahonen *et al.* and the moderate invasive mesenchymal tumor cells used in our studies. Another explanation may be differences in TIMP3 expression levels by adenoviral-based and lentiviral-based systems. This is supported by the fact that there is a correlation between the amount of TIMP3 and the apoptosis response in the different tested cell clones. Indeed, we detected high levels of TIMP3 in the supernatants of the overexpressing cells, indicating that the amount of endogenous TIMP3 is much higher than in the stimulation experiments with rhTIMP3.

Strikingly, the mechanism by which TIMP3 may directly induce apoptosis is a controversial issue as well. Studies on melanoma cells support the notion that adenoviral TIMP3 alone is able to induce apoptosis by its MMP-inhibitory activity, leading to accumulation, (auto) multimerization, and activation of death receptors even in the presence of limited amount of their ligands [23]. In contrast, induction of apoptosis in various other cancer cells, including a mesenchymal fibrosarcoma cell line, has shown to be independent of MMP inhibition und therefore suggested to be independent of death receptor signaling [19]. Likewise, lentiviral overexpression of TIMP3 by Cal78 cells led to a dramatic induction of death receptor ligand-independent apoptotic cell death, reflected by induction of caspase 3 and 7 activities up to 9-fold and cleavage of PARP and caspase-3 and -7 in these cells. Similar to adenoviral-based overexpression of TIMP3 in melanoma cells, overexpression of TIMP3 in Cal78 cells caused activation of caspase-8, a key mediator in death receptor-mediated signaling. This indicates that analog to melanoma cells, TIMP3 overexpression may lead to accumulation and subsequent multimerization and activation of death receptors [23].

TIMP3 is sequestered in the extracellular matrix by specifically interacting with sulfated glycosaminoglycans (GAGs) via its N-terminal domain [6] and matrix binding through the N-terminal domain is sufficient for the proapoptotic effect of TIMP3 in melanoma cells [23]. Although, the proapoptotic activity of TIMP3 has been mapped to the three N-terminal loops of the molecule necessary for the inhibition of MMP activity [24], it is also possible that TIMP3 promotes apoptosis by interfering with survival signals provided by the ECM to cells. This is supported by our observation, that lentiviral TIMP3 overexpression leads to a decrease in cRaf/Erk and Akt signaling. The phosphatidylinositol 3-kinase (PI3K)/AKT and the Ras/Raf/ERK signaling pathways are activated by many growth factors and cytokines and subsequently play critical roles in driving cell proliferation and preventing apoptosis [41], [42]. Many GAG chains are part of proteoglycan molecules attached to the cell surface and various cytokines and growth factors employ proteoglycans, such as syndecans or GPI-anchoraged glypicans as co-receptors [43]. In our study, withdrawal of serum caused a further increase in TIMP3 mediated apoptosis, indicating that activation of survival pathways by serum-derived factors highly influence TIMP3 induced apoptosis.

In this context, the observed downregulation of survival signals together with a reduced apoptosis response after shedding of proteoglycans with heparinase, suggest that binding of TIMP3 to cell surface proteoglycans may lead to impaired co-receptor function and subsequently inadequate survival signaling. Indeed, we observed a slight but significant decrease of apoptosis after EGF stimulation (figure 4D), supporting our hypothesis regarding the inhibition of co-receptor function by TIMP3. However, activation of more than one specific receptor may necessary to effectively counteract TIMP3-induced apoptosis.

Herein, we show that lentiviral expressed TIMP3 highly promotes ligand-dependent as well as -independent death receptor signaling in mesenchymal Cal78 cells. Moreover, TIMP3-overexpression leads to the blockade of survival signaling pathways. Most importantly, binding of TIMP3 to cell surface proteoglycans appears to be a prerequisite for the induction of apoptosis. In conclusion, the results provide for the first time, evidence that exclusively cell surface-bound TIMP3 induces apoptosis and that beside ligand-independent death receptor autoactivation, inhibition of survival pathways may contribute to apoptotic signaling.
